# Nursing Regulation Literature in Canada: Protocol for a Scoping Review

**DOI:** 10.2196/56163

**Published:** 2024-07-26

**Authors:** Patrick Chiu, Kathleen Leslie, Janice Y Kung

**Affiliations:** 1 Faculty of Nursing University of Alberta Edmonton, AB Canada; 2 Faculty of Health Disciplines Athabasca University Athabasca, AB Canada; 3 Geoffrey & Robyn Sperber Health Sciences Library University of Alberta Edmonton, AB Canada

**Keywords:** professional regulation, health profession regulation, nursing workforce, licensure, continuing competence, standards of practice, professional conduct, regulatory reform, nursing, Canada, health practitioner, nursing regulation, literature, practitioner regulation

## Abstract

**Background:**

Significant reforms are occurring in health practitioner regulation across Canada. Within the nursing profession, growing workforce challenges and health system demands have accelerated the pace of changes to nursing regulation policies and practices. There is significant political investment to modernize and harmonize nursing regulation across Canada, and evidence is needed to guide regulatory decision-making. To better understand the current state of scholarship and the gaps that exist, a comprehensive understanding of the available literature informing nursing regulation in Canada is first warranted.

**Objective:**

The objective of this scoping review is to examine the nature, extent, and range of literature focused on nursing regulation in Canada.

**Methods:**

The review will be conducted in accordance with the Joanna Briggs Institute guidelines for scoping reviews. We will search electronic databases, including Ovid MEDLINE, Ovid EMBASE, CINAHL, Scopus, and Web of Science Core Collection. We will also search for grey literature using the websites of Canadian nursing regulatory bodies, nursing organizations, and other leading Canadian regulatory organizations. No limitations will be placed on the year of publication. The review will include papers that explore nursing regulation in Canada, including topics such as education program accreditation or approval, licensure, standards of practice and code of conduct/ethics development and enforcement, continuing competence, discipline and conduct, regulatory models, governance, and reform. We will extract data using a predeveloped tool. Data will be analyzed using descriptive statistics and conventional content analysis.

**Results:**

A preliminary search in Ovid MEDLINE was undertaken on December 7, 2023, and a full search was conducted in 5 academic databases on March 15, 2024. Findings will be presented using evidence tables and a narrative summary. Reporting will follow the PRISMA-ScR (Preferred Reporting Items for Systematic reviews and Meta-Analyses extension for Scoping Reviews) guidelines. This scoping review is expected to be completed in early 2025.

**Conclusions:**

The results will be disseminated through conference presentations and a publication in a peer-reviewed journal. The findings will provide a comprehensive overview of the state of nursing regulation literature across Canada and inform the development of a focused research agenda.

**Trial Registration:**

Open Science Framework osf.io/3qk8t; https://osf.io/bm7jv

**International Registered Report Identifier (IRRID):**

DERR1-10.2196/56163

## Introduction

### Background

The primary goal of health practitioner regulation is to protect the public by ensuring the delivery of safe, competent, and ethical care [1]. While regulatory schemes differ across jurisdictional contexts, in general, health practitioner regulation across North America involves several core functions such as providing oversight on education programs, maintaining a public register of practitioners, developing and enforcing standards of practice and codes of conduct, and managing unprofessional conduct [2-5]. Health practitioner regulation plays a critical role in managing risk and reducing potential harm to the public. While regulatory bodies have historically focused their work at the individual practitioner level, scholars and regulatory experts increasingly recognize that regulation does not occur in a vacuum. Improving health practitioner regulatory systems also strongly contributes to health workforce planning and broader health system goals such as universal health coverage [4,6]. To improve health practitioner regulation, there is a need to better understand the breadth and depth of the evidence available to guide decision-making; identify knowledge gaps that exist; and examine the impacts and outcomes of regulation on practitioners, patients, and health systems.

Large-scale reviews conducted on the topic of health practitioner regulation provide a useful understanding of the current state of literature within a global context. For example, Browne et al [7] used a rapid evidence assessment to examine the international literature on health care and professional regulation. They found that the literature focuses on a variety of topics, including how regulators address harm prevention and patient safety, processes for addressing unprofessional conduct and complaints, ways that regulatory bodies address the quality of education and training, work related to the maintenance of registers, the development and impact of regulatory guidance, and the relationships between regulators and stakeholders. A recent large-scale international integrative review conducted by Leslie et al [4] explored the available evidence on the design, delivery, and effectiveness of health practitioner regulation to inform policy decisions. This review included many academic and grey literature sources and mapped the existing literature using a modified Donabedian framework that includes structures, processes, and outcomes.

Despite this body of scholarship, an overarching conclusion was that health practitioner regulation is a relatively underdeveloped field of academic study. Specifically, the existing evidence is diffuse, largely descriptive, and insufficient to support strong conclusions about the impact of different regulatory approaches, policies, and practices on patient safety, health workforce, and health system outcomes. Another important area of consideration is context, as regulatory schemes that work in one jurisdiction may not necessarily work in another given varied social, political, economic, and historical factors. As a result, more specific knowledge syntheses on health practitioner regulation at the profession or country level may be of particular use to guide decision-making and inform the development of policy and research agendas.

Our scan of Canadian nursing regulators’ websites illustrates that key priorities include the modernization of governance structures, amalgamation of regulatory bodies, separation of professional associations and regulatory bodies, alignment of principles of “right-touch regulation,” reduction of barriers to enable the integration of internationally educated nurses, and shifts in policy to enable the introduction of technology such as virtual care. In 2021, the National Council of State Boards of Nursing convened regulatory experts, including leaders of Canadian regulatory bodies, to create a global research agenda of nursing regulation. Stakeholders identified several research areas of interest, including labor mobility, regulation and governance, education, practice, continuing competency, discipline, and telehealth [8]. Although these priorities provide a blueprint for future research, there is first a need to fully understand the breadth and depth of available literature focused on nursing regulation in Canada.

A preliminary search on Ovid MEDLINE, the Cochrane Database of Systematic Reviews, and Joanna Briggs Institute (JBI) Evidence Synthesis database identified no current or in-progress systematic reviews or scoping reviews on this topic. Gaining a comprehensive understanding of the available evidence is critical for identifying knowledge gaps so that Canadian nursing stakeholders can invest their time and resources strategically to engage in high-impact research and knowledge development. To fill this gap, we chose to conduct a scoping review as our focus is on mapping the existing literature and providing an overview of evidence, concepts, and studies within the field of nursing regulation in Canada [9,10]. This protocol has been developed using the reporting guidelines in the PRISMA-P (Preferred Reporting Items for Systematic reviews and Meta-Analyses Protocols) as they apply to scoping reviews [11] (see Multimedia Appendix 1).

### Objectives and Review Questions

The overarching objective of this scoping review is to explore the breadth and depth of literature on nursing regulation in Canada. The research questions guiding our review are:

1. What is the nature, extent, and range of available scholarship informing nursing regulation within the Canadian context?
2. How does the extant scholarship align with emerging health practitioner regulation trends and what are the knowledge gaps that exist?

## Methods

### Design

We will conduct this scoping review in accordance with the JBI methodology for scoping reviews [12], drawing on Arksey and O’Malley’s [13] initial framework, which has been further enhanced by Levac et al [14] and Peters et al [15]. Our review will be organized into the following 6 stages: (1) identifying the research question and aligning it with the review objectives; (2) identifying relevant studies using inclusion and exclusion criteria that are aligned with the research objective and questions; (3) selecting relevant studies using a planned approach to evidence searching, selection, data extraction, and the presentation of evidence; (4) using both descriptive statistics and qualitative content analysis to chart the data; (5) consulting subject matter experts in regulation through professional networks; and (6) collating, summarizing, and reporting the evidence.

### Eligibility Criteria

#### Population

The population of interest includes Canadian nurses of all designations, including registered nurses, licensed practical nurses, registered psychiatric nurses, registered practical nurses (in Ontario), and nurse practitioners. Papers that discuss nursing regulation but focus more broadly on other regulated health professions (with no disaggregated data) will also be included to ensure the wide range of evidence and scholarship is captured.

#### Concept

The key concept of interest is professional regulation. We draw on the broad definition of professional regulation offered by Benton et al [2] (p. 307), where nursing regulation is defined as:

all those legitimate, appropriate and sustained means whereby order, identity, consistency, control and accountability are brought to practitioners through legally enforced, professional and/or voluntary action resulting in enhanced protection of the public, efficient and effective trans-jurisdictional movement, and the ongoing re-alignment of professional practice to patient and societal needs.

We will include papers that focus on any of the core functions of regulation, including education program accreditation or approval, registration and licensure, standards of practice and code of conduct/ethics development and enforcement, continuing competence, and discipline and conduct, as well as papers regarding topics such as regulatory models, governance, trade and mobility agreements, and reform.

#### Context

The review will include all relevant literature on nursing regulation within Canada. We chose to focus on this country as the purpose of the review is to inform the development of a research agenda to strengthen professional nursing regulation in Canada.

### Types of Sources

We will consider quantitative, mixed methods, and qualitative studies. Quantitative studies may include experimental and quasiexperimental study designs, including randomized controlled trials, nonrandomized controlled trials, before-and-after studies, and interrupted time-series studies; analytical observational studies, including prospective and retrospective cohort studies; case-control studies; analytical cross-sectional studies; and descriptive observational study designs, including case series, individual case reports, and descriptive cross-sectional studies. Qualitative studies may include but are not limited to designs such as phenomenology, grounded theory, ethnography, qualitative description, interpretive description, action research, and feminist research. Systematic reviews will be excluded; however, reference lists will be screened for relevant studies. Discussion papers, commentaries, and opinion papers will also be considered for inclusion if they provide substantive exploration, examination, or critique of nursing regulation in Canada. We will search for grey literature such as policy documents and reports from the websites of Canadian organizations relevant to nursing regulation. Theses will be included given the potential for in-depth exploration of nursing regulatory issues. Books and unavailable articles will be excluded. See Multimedia Appendix 2 for the detailed inclusion and exclusion criteria.

### Search Strategy

Our search strategy aims to locate both published and unpublished studies. A professional research librarian was consulted throughout the development of the search strategy. An initial limited search of Ovid MEDLINE was undertaken to identify articles on our review topic (see Multimedia Appendix 3). The search strategy, including all identified keywords and index terms, was adapted for each included database or information source. The databases searched include Ovid MEDLINE, Ovid EMBASE, CINAHL, Scopus, and Web of Science Core Collection (see Multimedia Appendix 4). We will screen the reference lists of all included articles to identify additional papers. Given our focus on identifying the nature, extent, and range of scholarship focused on nursing regulation in Canada, we will not place any limitations on the date of publication. Non-English papers will be excluded due to resource constraints. We will search for grey literature using the websites of Canadian nursing regulatory bodies, the Canadian Nurses Association, the Canadian Association of Schools of Nursing, the Canadian Nurses Protective Society, the Canadian Network of Agencies of Regulation, and newsletters and legal updates from law firms focused on professional regulation in Canada. Further, we will search the first 200 citations from Google Scholar.

### Study/Source of Evidence Selection

Following the search, we will collate and upload all identified citations into Covidence, a review management software system [16], and remove duplicates. We chose to use Covidence for this purpose as it enables collaboration among multiple reviewers. Following a pilot test using 10% of the studies, titles and abstracts will be screened by 2 or more independent reviewers for assessment against the inclusion criteria for the review. Potentially relevant sources will be retrieved in full text. The full text of selected citations will be assessed in detail against the inclusion criteria by 2 independent reviewers. Reasons for excluding sources during full-text review will be recorded and reported in the scoping review. Any disagreements that arise between the reviewers at each stage of the selection process will be resolved by a third reviewer. Citations of included studies will be uploaded into Zotero, a reference manager software program. The results of the search and the selection process will be reported in full in the final scoping review, guided by the PRISMA-ScR (Preferred Reporting Items for Systematic reviews and Meta-Analyses Extension for Scoping Review) checklist [17].

### Data Extraction

Data will be extracted from papers included in the scoping review by 2 independent reviewers using a data extraction tool developed by the reviewers. The data extracted will include the year of publication, type of publication, focus and or/aim of the paper, nursing designation, jurisdiction (eg, international, national, provincial, territorial), and key findings/concepts. We will group the focus or aim of each paper using the core regulatory functions of education program and accreditation, registration or licensure, standards of practice or code of ethics, continuing competence, discipline, and conduct. Given significant changes within the health professions regulatory landscape in Canada and the likelihood of scholarship on this topic, we will also group papers under categories such as governance, reform, regulatory models, and trade and mobility agreements. A draft extraction form is provided in Multimedia Appendix 5. The draft data extraction tool will be modified and revised as necessary during data extraction. We will pilot the extraction form using 10% of the included articles and resolve any discrepancies through discussion. Modifications from the protocol will be detailed in the scoping review. If appropriate and when required, authors of papers will be contacted to request missing or additional data. Critical appraisal of individual sources of evidence will not be completed as this is generally not required for scoping reviews.

### Data Analysis

We will organize results using evidence tables to ensure the data are presented in a clear and structured format. We will use descriptive statistics to illustrate frequency counts and percentages for data such as year of publication, type of publication, areas of focus, type of nursing designations discussed, and jurisdictions of focus. Conventional content analysis will be used to identify and map the key purpose, aims, findings, and concepts within the included papers. Conventional content analysis [18] is an appropriate analytical method as it enables the development of broader categories based on coded data. A narrative summary will accompany the evidence tables and describe how the results relate to the review objective and questions. To ensure the overall reporting quality of this scoping review, reporting will follow the PRISMA-ScR guidelines. All retrieved records from the search strategy, included and excluded records from primary and secondary screening, and records retrieved from other sources will be reported using an adapted PRISMA flow diagram to enhance the reproducibility of this scoping review.

## Results

This review was registered with the Open Science Framework on January 8, 2024. A preliminary search in Ovid MEDLINE was undertaken on December 7, 2023. After confirming the search terms, the medical librarian (JYK) performed a full search on March 15, 2024, in the Ovid MEDLINE, Ovid EMBASE, CINAHL, Scopus, and Web of Science Core Collection databases. A total of 1850 records were retrieved and when all duplicates were removed, 951 unique results remained for the initial title and abstract screening. We anticipate completing the scoping review in early 2025.

## Discussion

### Projected Significance and Contributions

Health practitioner regulation across Canada is changing rapidly, with legislative reforms occurring in several provinces giving rise to new regulatory and governance schemes. Notably, in British Columbia, the enactment of the Health Professions and Occupations Act in 2022—one of the most significant regulatory reforms in Canada’s history—paved the way for a new regulatory scheme for health practitioner regulation within the province [19]. These changes have already influenced regulatory reform in other Canadian jurisdictions such as Nova Scotia, with Bill 323, An Act to Provide a Common Legislative Foundation for Regulated Health Professions [20]. Nursing regulation in particular has been thrust into the spotlight since the COVID-19 pandemic [21]. With the combination of vexing issues such as increasing public and government mistrust, labor market challenges, the introduction of new ways of working, and the explosion of digital technologies, nursing regulators continue to face pressures to modernize and reform their regulatory approaches to ensure they are fit for purpose and responsive to today’s health system needs. The appointment of a federal chief nursing officer and growing political interest and investment in reforming nursing regulation [22] are strong determinants for continued change.

To our knowledge, this is the first scoping review that will examine the state of Canadian nursing regulation literature. We anticipate that the amount of evidence available to support regulatory decision-making will be relatively low. Given historical and contemporary health system trends and issues, we hypothesize that the majority of the literature will be focused on licensure and registration policies and practices for both Canadian and internationally educated nurses. Further, we anticipate that the majority of the literature will be situated in provinces with a larger population and nursing workforce. Having a comprehensive understanding of the existing body of work will provide a meaningful foundation to create a research agenda to inform the advancement of nursing regulation across Canada. We plan to disseminate our findings to our existing network of nursing regulation leaders through forums such as the Canadian Nurse Regulator Collaborative, conference presentations, and a peer-reviewed manuscript.

### Strengths and Limitations

Given the purpose of the scoping review, the quality of the literature will not be assessed. Further, due to significant reforms across Canada and ongoing changes to regulators’ websites, it may not be possible to capture all the relevant grey literature that has been produced. However, our comprehensive search strategy will provide a fulsome understanding of the nature, extent, and range of literature to identify research gaps and inform future areas of inquiry that can strengthen regulatory decision-making.

## Appendix

Multimedia Appendix 1 PRISMA-P (Preferred Reporting Items for Systematic reviews and Meta-Analyses Protocols) checklist.

Multimedia Appendix 2 Inclusion/exclusion screening form.

Multimedia Appendix 3 Ovid MEDLINE search strategy.

Multimedia Appendix 4 Full search strategies.

Multimedia Appendix 5 Data extraction instrument.

## Abbreviations

JBI: Joanna Briggs Institute
PRISMA-ScR: Preferred Reporting Items for Systematic reviews and Meta-Analyses extension for Scoping Reviews
PRISMA-P: Preferred Reporting Items for Systematic reviews and Meta-Analyses Protocols
