# Supplementary material for: Nursing Regulation Literature in Canada: Protocol for a Scoping Review
Source: JMIR Res Protoc. 2024 Jul 26;13:e56163. doi: 10.2196/56163 (PMC11316164; doi:10.2196/56163)
Supplement: Multimedia Appendix 2 [file resprot_v13i1e56163_app2.pdf]

Multimedia Appendix 2: Inclusion/Exclusion Screening Form

Criteria	Process
1. **Language**	The full text is available in English.<br>• If no, exclude based on language.<br>• If yes, move to criteria 2.
2. **Sources**	The paper is either primary research (quantitative, qualitative, mixed methods), commentaries or opinion papers, or grey literature (documents and reports from nursing and/or regulatory organizations in Canada).<br>• If no, exclude based on source.<br>• If yes, move to criteria 3.
3. **Geographical location (Context)**	The paper is focused on the Canadian context.<br>• If no, exclude based on geographical location.<br>• If yes, move to criteria 4.
4. **Population**	The paper focuses on nurses (registered nurses, registered psychiatric nurses, licensed practical nurses, nurse practitioners) or health care professionals including nurses.<br>• If no, exclude based on population.<br>• If yes, move to criteria 5.
5. **Concept**	The paper focuses on topics related to nursing regulation in Canada (i.e., education or program approval, licensure, standards of practice or code of ethics, continuing competence, discipline and conduct, regulatory models, governance, and reform).<br>• If no, exclude based on concept.<br>• If yes, include.
