# Supplementary material for: Nursing Regulation Literature in Canada: Protocol for a Scoping Review
Source: JMIR Res Protoc. 2024 Jul 26;13:e56163. doi: 10.2196/56163 (PMC11316164; doi:10.2196/56163)
Supplement: Multimedia Appendix 3 [file resprot_v13i1e56163_app3.pdf]

Multimedia Appendix 3: OVID MEDLINE Search Strategy

#	Query	Results from Dec 7 2023
1	(nurs* adj4 (registration* or regulat* or licensing or licensure)).mp.	8036
2	exp Licensure, Nursing/ 4680	4680
3	1 or 2	8036
4	(Canad* or British Columbia or Colombie Britannique or Alberta* or Saskatchewan or Manitoba* or Ontario or Quebec or (New Brunswick not New Jersey) or Nouveau Brunswick or Nova Scotia or Nouvelle Ecosse or Prince Edward Island or Newfoundland or Labrador or Nunavut or NWT or Northwest Territories or Yukon or Nunavik or Inuvialuit).mp,jw,nw. or (Abbotsford or Airdrie or Ajax or Aurora or Barrie or Belleville or Blainville or Brampton or Brantford or Brossard or Burlington or Burnaby or Caledon or Calgary or Cape Breton or Chatham Kent or Chilliwack or Clarington or Coquitlam or Drummondville or Edmonton or Fredericton or Fort McMurray or Gatineau or Granby or Grande Prairie or Sudbury or Guelph or Halton Hills or Iqaluit or Inuvik or Kamloops or Kawartha Lakes or Kelowna or Kingston or Kitchener or Langley or Laval or Lethbridge or Levis or Longueuil or Maple Ridge or Markham or Medicine Hat or Milton or Mirabel or Mississauga or Moncton or Montreal or Nanaimo or New Westminster or Newmarket or Niagara Falls or Norfolk County or North Bay or North Vancouver or Oakville or Oshawa or Ottawa or Peterborough or Pickering or Port Coquitlam or Prince George or Quebec City or Red Deer or Regina or Repentigny or Richmond or Richmond Hill or Saanich or Saguenay or Saint John or Saint-Hyacinthe or Saint-Jean-sur-Richelieu or Saint-Jerome or Sarnia or Saskatoon or Sault Ste Marie or Sherbrooke or St Albert or St Catharines or St John's or Strathcona County or Surrey or Terrebonne or Thunder Bay or Toronto or Trois-Rivieres or Vancouver or Vaughan or ((Cambridge or (Halifax or Hamilton or London or Victoria or Waterloo or Welland or Whitby or Windsor)) not (UK or Britain or United Kingdom or England or Australia)) or Whitehorse or Winnipeg or Wood Buffalo or Yellowknife).ti,ab,kw.	592080
5	("College of Registered Psychiatric Nurses of British Columbia" or "College of Licensed Practical Nurses of British Columbia" or "College of Registered Nurses of British Columbia" or "British Columbia College of Nursing Professionals" or "British Columbia College of Nursing Professionals and Midwives" or "College of Registered Nurses of Alberta" or "College of Licensed Practical Nurses of Alberta" or "College of Registered Psychiatric Nurses of Alberta" or "College and Association Registered Nurses of Alberta" or "Saskatchewan Registered Nurses Association" or "Saskatchewan Association of Licensed Practical Nurses"	69

	or "Registered Psychiatric Nurses Association of Saskatchewan" or "College of Registered Nurses of Saskatchewan" or "College of Registered Nurses of Manitoba" or "College of Licensed Practical Nurses of Manitoba" or "College of Registered Psychiatric Nurses of Manitoba" or "College of Nurses of Ontario" or "Ordre des infirmieres et infirmiers du Quebec" or "Ordre des infirmieres et infirmiers auxiliaires du Quebec" or "Order of Nurses of Quebec" or "Quebec Order of Nurses" or "Nurses Association of New Brunswick" or "Association of New Brunswick Licensed Practical Nurses" or "Nova Scotia College of Nursing" or "College of Registered Nurses of Nova Scotia" or "College of Licensed Practical Nurses of Nova Scotia" or "College of Registered Nurses of Prince Edward Island" or "College of Licensed Practical Nurses of Prince Edward Island" or "College of Registered Nurses of Newfoundland and Labrador" or "College of Licensed Practical Nurses of Newfoundland and Labrador" or "Association of Registered Nurses of Newfoundland and Labrador" or "Registered Nurses Association of the Northwest Territories and Nunavut" or "Yukon Registered Nurses Association").mp.	
6	4 or 5	592080
7	3 and 6	446
8	"journal of nursing regulation".jn.	37
9	4 and 8	1
10	7 or 9	446
11	limit 10 to English	424
