# Supplementary material for: Nursing Regulation Literature in Canada: Protocol for a Scoping Review
Source: JMIR Res Protoc. 2024 Jul 26;13:e56163. doi: 10.2196/56163 (PMC11316164; doi:10.2196/56163)
Supplement: Multimedia Appendix 4 [file resprot_v13i1e56163_app4.pdf]

Multimedia Appendix 4: Full Search Strategies

The medical librarian (JYK) developed and executed comprehensive searches in Ovid MEDLINE, Ovid Embase, CINAHL, Scopus, and Web of Science Core Collection on March 15, 2024.

Database	Search Strategy
**MEDLINE**<br><br>Ovid MEDLINE(R) ALL 1946 to March 14, 2024	1. (nurs* adj4 (registration* or regulat* or licensing or licensure)).mp.<br>2. exp Licensure, Nursing/<br>3. 1 or 2<br>4. (Canad* or British Columbia or Colombie Britannique or Alberta* or Saskatchewan or Manitoba* or Ontario or Quebec or (New Brunswick not New Jersey) or Nouveau Brunswick or Nova Scotia or Nouvelle Ecosse or Prince Edward Island or Newfoundland or Labrador or Nunavut or NWT or Northwest Territories or Yukon or Nunavik or Inuvialuit).mp,jw,nw. or (Abbotsford or Airdrie or Ajax or Aurora or Barrie or Belleville or Blainville or Brampton or Brantford or Brossard or Burlington or Burnaby or Caledon or Calgary or Cape Breton or Chatham Kent or Chilliwack or Clarington or Coquitlam or Drummondville or Edmonton or Fredericton or Fort McMurray or Gatineau or Granby or Grande Prairie or Sudbury or Guelph or Halton Hills or Iqaluit or Inuvik or Kamloops or Kawartha Lakes or Kelowna or Kingston or Kitchener or Langley or Laval or Lethbridge or Levis or Longueuil or Maple Ridge or Markham or Medicine Hat or Milton or Mirabel or Mississauga or Moncton or Montreal or Nanaimo or New Westminster or Newmarket or Niagara Falls or Norfolk County or North Bay or North Vancouver or Oakville or Oshawa or Ottawa or Peterborough or Pickering or Port Coquitlam or Prince George or Quebec City or Red Deer or Regina or Repentigny or Richmond or Richmond Hill or Saanich or Saguenay or Saint John or Saint-Hyacinthe or Saint-Jean-sur-Richelieu or Saint-Jerome or Sarnia or Saskatoon or Sault Ste Marie or Sherbrooke or St Albert or St Catharines or St John's or Strathcona County or Surrey or Terrebonne or Thunder Bay or Toronto or Trois-Rivieres or Vancouver or Vaughan or ((Cambridge or (Halifax or Hamilton or London or Victoria or Waterloo or Welland or Whitby or Windsor)) not (UK or Britain or United Kingdom or England or Australia)) or Whitehorse or Winnipeg or Wood Buffalo or Yellowknife).ti,ab,kw.<br>5. ("College of Registered Psychiatric Nurses of British Columbia" or "College of Licensed Practical Nurses of British Columbia" or "College of Registered Nurses of British Columbia" or "British Columbia College of Nursing Professionals" or "British Columbia College of Nursing Professionals and Midwives" or "College of Registered Nurses of Alberta" or "College of Licensed Practical Nurses of Alberta" or "College of Registered Psychiatric Nurses of Alberta" or "College and Association Registered Nurses of Alberta" or "Saskatchewan Registered Nurses Association" or "Saskatchewan Association of Licensed

	Practical Nurses" or "Registered Psychiatric Nurses Association of Saskatchewan" or "College of Registered Nurses of Saskatchewan" or "College of Registered Nurses of Manitoba" or "College of Licensed Practical Nurses of Manitoba" or "College of Registered Psychiatric Nurses of Manitoba" or "College of Nurses of Ontario" or "Ordre des infirmieres et infirmiers du Quebec" or "Ordre des infirmieres et infirmiers auxiliaires du Quebec" or "Order of Nurses of Quebec" or "Quebec Order of Nurses" or "Nurses Association of New Brunswick" or "Association of New Brunswick Licensed Practical Nurses" or "Nova Scotia College of Nursing" or "College of Registered Nurses of Nova Scotia" or "College of Licensed Practical Nurses of Nova Scotia" or "College of Registered Nurses of Prince Edward Island" or "College of Licensed Practical Nurses of Prince Edward Island" or "College of Registered Nurses of Newfoundland and Labrador" or "College of Licensed Practical Nurses of Newfoundland and Labrador" or "Association of Registered Nurses of Newfoundland and Labrador" or "Registered Nurses Association of the Northwest Territories and Nunavut" or "Yukon Registered Nurses Association").mp.<br>6. 4 or 5<br>7. 3 and 6<br>8. "journal of nursing regulation".jn.<br>9. 4 and 8<br>10. 7 or 9<br>11. limit 10 to english
**Embase**<br><br>Ovid Embase 1974 to 2024 March 14	1. (nurs* adj4 (registration* or regulat* or licensing or licensure)).mp.<br>2. (Canad* or British Columbia or Colombie Britannique or Alberta* or Saskatchewan or Manitoba* or Ontario or Quebec or (New Brunswick not New Jersey) or Nouveau Brunswick or Nova Scotia or Nouvelle Ecosse or Prince Edward Island or Newfoundland or Labrador or Nunavut or NWT or Northwest Territories or Yukon or Nunavik or Inuvialuit).mp,jx. or (Abbotsford or Airdrie or Ajax or Aurora or Barrie or Belleville or Blainville or Brampton or Brantford or Brossard or Burlington or Burnaby or Caledon or Calgary or Cape Breton or Chatham Kent or Chilliwack or Clarington or Coquitlam or Drummondville or Edmonton or Fredericton or Fort McMurray or Gatineau or Granby or Grande Prairie or Sudbury or Guelph or Halton Hills or Iqaluit or Inuvik or Kamloops or Kawartha Lakes or Kelowna or Kingston or Kitchener or Langley or Laval or Lethbridge or Levis or Longueuil or Maple Ridge or Markham or Medicine Hat or Milton or Mirabel or Mississauga or Moncton or Montreal or Nanaimo or New Westminster or Newmarket or Niagara Falls or Norfolk County or North Bay or North Vancouver or Oakville or Oshawa or Ottawa or Peterborough or Pickering or Port Coquitlam or Prince George or Quebec City or Red Deer or Regina or Repentigny or Richmond or Richmond Hill or Saanich or Saguenay or Saint John or Saint-Hyacinthe or Saint-Jean-sur-Richelieu or Saint-Jerome or Sarnia or Saskatoon or Sault Ste Marie or Sherbrooke or St Albert or St Catharines or St John's or Strathcona County or Surrey or Terrebonne

	or Thunder Bay or Toronto or Trois-Rivieres or Vancouver or Vaughan or ((Cambridge or (Halifax or Hamilton or London or Victoria or Waterloo or Welland or Whitby or Windsor)) not (UK or Britain or United Kingdom or England or Australia)) or Whitehorse or Winnipeg or Wood Buffalo or Yellowknife).ti,ab,kw.<br>3. ("College of Registered Psychiatric Nurses of British Columbia" or "College of Licensed Practical Nurses of British Columbia" or "College of Registered Nurses of British Columbia" or "British Columbia College of Nursing Professionals" or "British Columbia College of Nursing Professionals and Midwives" or "College of Registered Nurses of Alberta" or "College of Licensed Practical Nurses of Alberta" or "College of Registered Psychiatric Nurses of Alberta" or "College and Association Registered Nurses of Alberta" or "Saskatchewan Registered Nurses Association" or "Saskatchewan Association of Licensed Practical Nurses" or "Registered Psychiatric Nurses Association of Saskatchewan" or "College of Registered Nurses of Saskatchewan" or "College of Registered Nurses of Manitoba" or "College of Licensed Practical Nurses of Manitoba" or "College of Registered Psychiatric Nurses of Manitoba" or "College of Nurses of Ontario" or "Ordre des infirmieres et infirmiers du Quebec" or "Ordre des infirmieres et infirmiers auxiliaires du Quebec" or "Order of Nurses of Quebec" or "Quebec Order of Nurses" or "Nurses Association of New Brunswick" or "Association of New Brunswick Licensed Practical Nurses" or "Nova Scotia College of Nursing" or "College of Registered Nurses of Nova Scotia" or "College of Licensed Practical Nurses of Nova Scotia" or "College of Registered Nurses of Prince Edward Island" or "College of Licensed Practical Nurses of Prince Edward Island" or "College of Registered Nurses of Newfoundland and Labrador" or "College of Licensed Practical Nurses of Newfoundland and Labrador" or "Association of Registered Nurses of Newfoundland and Labrador" or "Registered Nurses Association of the Northwest Territories and Nunavut" or "Yukon Registered Nurses Association").mp.<br>4. 2 or 3<br>5. 1 and 4<br>6. limit 5 to english language
**CINAHL**	S1 nurs* N4 (registration* or regulat* or licensing or licensure)<br>S2 (MH "Licensure, Nursing")<br>S3 S1 OR S2<br>S4 Canad* or "British Columbia" or "Colombie Britannique" or Alberta* or Saskatchewan or Manitoba* or Ontario or Quebec or ("New Brunswick" not "New Jersey") or "Nouveau Brunswick" or "Nova Scotia" or "Nouvelle Ecosse" or "Prince Edward Island" or Newfoundland or Labrador or Nunavut or NWT or "Northwest Territories" or Yukon or Nunavik or Inuvialuit or Abbotsford or Airdrie or Ajax or Aurora or Barrie or Belleville or Blainville or Brampton or Brantford or Brossard or Burlington or Burnaby or Caledon or Calgary or Cambridge or "Cape Breton" or Chatham or Kent or Chilliwack or Clarington or Coquitlam or Drummondville or

		Edmonton or "Fort McMurray" or Fredericton or Gatineau or Granby or "Grande Prairie" or Sudbury or Guelph or "Halton Hills" or Iqaluit or Inuvik or Kamloops or "Kawartha Lakes" or Kelowna or Kingston or Kitchener or Langley or Laval or Lethbridge or Levis or Longueuil or "Maple Ridge" or Markham or "Medicine Hat" or Milton or Mirabel or Mississauga or Moncton or Montreal or Nanaimo or "New Westminster" or Newmarket or "Niagara Falls" or "Norfolk County" or "North Bay" or "North Vancouver" or Oakville or Oshawa or Ottawa or Peterborough or Pickering or "Port Coquitlam" or "Prince George" or "Quebec City" or "Red Deer" or Regina or Repentigny or (Richmond not Virginia) or "Richmond Hill" or Saanich or Saguenay or "Saint John" or "Saint-Hyacinthe" or "Saint-Jean-sur-Richelieu" or "Saint-Jerome" or Sarnia or Saskatoon or "Sault Ste Marie" or Sherbrooke or "St Albert" or "St Catharines" or "St John's" or "Strathcona County" or Surrey or Terrebonne or "Thunder Bay" or Toronto or "Trois-Rivieres" or Vancouver or Vaughan or ((Halifax or Hamilton or London or Victoria or Waterloo or Welland or Whitby or Windsor) not (UK or "United Kingdom" or Britain or England or Australia)) or Whitehorse or Winnipeg or "Wood Buffalo" or Yellowknife Show Less
	S5	"College of Registered Psychiatric Nurses of British Columbia" or "College of Licensed Practical Nurses of British Columbia" or "College of Registered Nurses of British Columbia" or "British Columbia College of Nursing Professionals" or "British Columbia College of Nursing Professionals and Midwives" or "College of Registered Nurses of Alberta" or "College of Licensed Practical Nurses of Alberta" or "College of Registered Psychiatric Nurses of Alberta" or "College and Association Registered Nurses of Alberta" or "Saskatchewan Registered Nurses Association" or "Saskatchewan Association of Licensed Practical Nurses" or "Registered Psychiatric Nurses Association of Saskatchewan" or "College of Registered Nurses of Saskatchewan" or "College of Registered Nurses of Manitoba" or "College of Licensed Practical Nurses of Manitoba" or "College of Registered Psychiatric Nurses of Manitoba" or "College of Nurses of Ontario" or "Ordre des infirmieres et infirmiers du Quebec" or "Ordre des infirmieres et infirmiers auxiliaires du Quebec" or "Order of Nurses of Quebec" or "Quebec Order of Nurses" or "Nurses Association of New Brunswick" or "Association of New Brunswick Licensed Practical Nurses" or "Nova Scotia College of Nursing" or "College of Registered Nurses of Nova Scotia" or "College of Licensed Practical Nurses of Nova Scotia" or "College of Registered Nurses of Prince Edward Island" or "College of Licensed Practical Nurses of Prince Edward Island" or "College of Registered Nurses of Newfoundland and Labrador" or "College of Licensed Practical Nurses of Newfoundland and Labrador" or "Association of Registered Nurses of Newfoundland and Labrador" or "Registered Nurses Association of the Northwest Territories and Nunavut" or "Yukon Registered Nurses Association" Show Less
	S6	S4 OR S5

	S7 S3 AND S6<br>S8 SO "journal of nursing regulation"<br>S9 S4 AND S8<br>S10 S7 OR S9<br>S11 S7 OR S9 (Limiters: English language)
**Scopus**	TITLE-ABS-KEY (nurs* W/4 (registration* OR regulat* OR licensing OR licensure)) AND ((TITLE-ABS-KEY (canad* OR "British Columbia" OR "Colombie Britannique" OR alberta* OR saskatchewan OR manitoba* OR ontario OR quebec OR "New Brunswick" OR "Nouveau Brunswick" OR "Nova Scotia" OR "Nouvelle Ecosse" OR "Prince Edward Island" OR newfoundland OR labrador OR nunavut OR nwt OR "Northwest Territories" OR yukon OR nunavik OR inuvialuit OR abbotsford OR airdrie OR ajax OR aurora OR barrie OR belleville OR blainville OR brampton OR brantford OR brossard OR burlington OR burnaby OR caledon OR calgary OR cambridge OR "Cape Breton" OR chatham OR kent OR chilliwack OR clarington OR coquitlam OR drummondville OR edmonton OR "Fort McMurray" OR fredericton OR gatineau OR granby OR "Grande Prairie" OR sudbury OR guelph OR "Halton Hills" OR iqaluit OR inuvik OR kamloops OR "Kawartha Lakes" OR kelowna OR kingston OR kitchener OR langley OR laval OR lethbridge OR levis OR longueuil OR "Maple Ridge" OR markham OR "Medicine Hat" OR milton OR mirabel OR mississauga OR moncton OR montreal OR nanaimo OR "New Westminster" OR newmarket OR "Niagara Falls" OR "Norfolk County" OR "North Bay" OR "North Vancouver" OR oakville OR oshawa OR ottawa OR peterborough OR pickering OR "Port Coquitlam" OR "Prince George" OR "Quebec City" OR "Red Deer" OR regina OR repentigny OR richmond OR "Richmond Hill" OR saanich OR saguenay OR "Saint John" OR "Saint-Hyacinthe" OR "Saint-Jean-sur-Richelieu" OR "Saint-Jerome" OR sarnia OR saskatoon OR "Sault Ste Marie" OR sherbrooke OR "St Albert" OR "St Catharines" OR "St John's" OR "Strathcona County" OR surrey OR terrebonne OR "Thunder Bay" OR toronto OR "Trois-Rivieres" OR vancouver OR vaughan OR halifax OR hamilton OR london OR victoria OR waterloo OR welland OR whitby OR windsor OR whitehorse OR winnipeg OR "Wood Buffalo" OR yellowknife) AND NOT (TITLE-ABS-KEY (uk OR "United Kingdom" OR britain OR england OR australia OR "United States" OR virginia OR "New Jersey"))) OR TITLE-ABS-KEY ("College of Registered Psychiatric Nurses of British Columbia" OR "College of Licensed Practical Nurses of British Columbia" OR "College of Registered Nurses of British Columbia" OR "British Columbia College of Nursing Professionals" OR "British Columbia College of Nursing Professionals and Midwives" OR "College of Registered Nurses of Alberta" OR "College of Licensed Practical Nurses of Alberta" OR "College of Registered Psychiatric Nurses of Alberta" OR "College and Association Registered Nurses of Alberta" OR "Saskatchewan Registered Nurses Association" OR "Saskatchewan Association of Licensed Practical Nurses" OR "Registered Psychiatric Nurses Association of Saskatchewan" OR "College of Registered Nurses of Saskatchewan" OR "College of Registered Nurses of Manitoba" OR "College of Licensed Practical Nurses of Manitoba" OR

	"College of Registered Psychiatric Nurses of Manitoba" OR "College of Nurses of Ontario" OR "Ordre des infirmieres et infirmiers du Quebec" OR "Ordre des infirmieres et infirmiers auxiliaires du Quebec" OR "Order of Nurses of Quebec" OR "Quebec Order of Nurses" OR "Nurses Association of New Brunswick" OR "Association of New Brunswick Licensed Practical Nurses" OR "Nova Scotia College of Nursing" OR "College of Registered Nurses of Nova Scotia" OR "College of Licensed Practical Nurses of Nova Scotia" OR "College of Registered Nurses of Prince Edward Island" OR "College of Licensed Practical Nurses of Prince Edward Island" OR "College of Registered Nurses of Newfoundland and Labrador" OR "College of Licensed Practical Nurses of Newfoundland and Labrador" OR "Association of Registered Nurses of Newfoundland and Labrador" OR "Registered Nurses Association of the Northwest Territories and Nunavut" OR "Yukon Registered Nurses Association")) AND (LIMIT-TO (LANGUAGE , "English"))
**Web of Science Core Collection**<br><br>**&**<br><br>**ProQuest ™ Dissertations & Theses Citation Index**<br><br>(via Clarivate)	TS=(nurs* NEAR/4 (registration* OR regulat* OR licensing OR licensure)) AND ((TS=(canad* OR "British Columbia" OR "Colombie Britannique" OR alberta* OR saskatchewan OR manitoba* OR ontario OR quebec OR "New Brunswick" OR "Nouveau Brunswick" OR "Nova Scotia" OR "Nouvelle Ecosse" OR "Prince Edward Island" OR newfoundland OR labrador OR nunavut OR nwt OR "Northwest Territories" OR yukon OR nunavik OR inuvialuit OR abbotsford OR airdrie OR ajax OR aurora OR barrie OR belleville OR blainville OR brampton OR brantford OR brossard OR burlington OR burnaby OR caledon OR calgary OR cambridge OR "Cape Breton" OR chatham OR kent OR chilliwack OR clarington OR coquitlam OR drummondville OR edmonton OR "Fort McMurray" OR fredericton OR gatineau OR granby OR "Grande Prairie" OR sudbury OR guelph OR "Halton Hills" OR iqaluit OR inuvik OR kamloops OR "Kawartha Lakes" OR kelowna OR kingston OR kitchener OR langley OR laval OR lethbridge OR levis OR longueuil OR "Maple Ridge" OR markham OR "Medicine Hat" OR milton OR mirabel OR mississauga OR moncton OR montreal OR nanaimo OR "New Westminster" OR newmarket OR "Niagara Falls" OR "Norfolk County" OR "North Bay" OR "North Vancouver" OR oakville OR oshawa OR ottawa OR peterborough OR pickering OR "Port Coquitlam" OR "Prince George" OR "Quebec City" OR "Red Deer" OR regina OR repentigny OR richmond OR "Richmond Hill" OR saanich OR saguenay OR "Saint John" OR "Saint-Hyacinthe" OR "Saint-Jean-sur-Richelieu" OR "Saint-Jerome" OR sarnia OR saskatoon OR "Sault Ste Marie" OR sherbrooke OR "St Albert" OR "St Catharines" OR "St John's" OR "Strathcona County" OR surrey OR terrebonne OR "Thunder Bay" OR toronto OR "Trois-Rivieres" OR vancouver OR vaughan OR halifax OR hamilton OR london OR victoria OR waterloo OR welland OR whitby OR windsor OR whitehorse OR winnipeg OR "Wood Buffalo" OR yellowknife) NOT (TS=(uk OR "United Kingdom" OR britain OR england OR australia OR "United States" OR virginia OR "New Jersey"))) OR TS=("College of Registered Psychiatric Nurses of British Columbia" OR "College of Licensed Practical Nurses of British Columbia" OR "College of Registered Nurses of British Columbia" OR "British Columbia

	College of Nursing Professionals" OR "British Columbia College of Nursing Professionals and Midwives" OR "College of Registered Nurses of Alberta" OR "College of Licensed Practical Nurses of Alberta" OR "College of Registered Psychiatric Nurses of Alberta" OR "College and Association Registered Nurses of Alberta" OR "Saskatchewan Registered Nurses Association" OR "Saskatchewan Association of Licensed Practical Nurses" OR "Registered Psychiatric Nurses Association of Saskatchewan" OR "College of Registered Nurses of Saskatchewan" OR "College of Registered Nurses of Manitoba" OR "College of Licensed Practical Nurses of Manitoba" OR "College of Registered Psychiatric Nurses of Manitoba" OR "College of Nurses of Ontario" OR "Ordre des infirmieres et infirmiers du Quebec" OR "Ordre des infirmieres et infirmiers auxiliaires du Quebec" OR "Order of Nurses of Quebec" OR "Quebec Order of Nurses" OR "Nurses Association of New Brunswick" OR "Association of New Brunswick Licensed Practical Nurses" OR "Nova Scotia College of Nursing" OR "College of Registered Nurses of Nova Scotia" OR "College of Licensed Practical Nurses of Nova Scotia" OR "College of Registered Nurses of Prince Edward Island" OR "College of Licensed Practical Nurses of Prince Edward Island" OR "College of Registered Nurses of Newfoundland and Labrador" OR "College of Licensed Practical Nurses of Newfoundland and Labrador" OR "Association of Registered Nurses of Newfoundland and Labrador" OR "Registered Nurses Association of the Northwest Territories and Nunavut" OR "Yukon Registered Nurses Association"))<br><br>Refined by Languages: English
