# Supplementary material for: Nursing Regulation Literature in Canada: Protocol for a Scoping Review
Source: JMIR Res Protoc. 2024 Jul 26;13:e56163. doi: 10.2196/56163 (PMC11316164; doi:10.2196/56163)
Supplement: Multimedia Appendix 5 [file resprot_v13i1e56163_app5.pdf]

**Multimedia Appendix 5: Data Extraction Instrument**

| ID | Author | Year | Type of publication | Purpose and Aim | Focus in relation to core regulatory functions | Population | Jurisdiction | Key findings or concepts |
|---|---|---|---|---|---|---|---|---|
| | | | Quantitative<br><br>Qualitative<br><br>Commentaries and opinion papers<br><br>Discussion papers<br><br>Theses<br><br>Grey literature | | Education program approval and accreditation<br><br>Registration or licensure<br><br>Standards of practice or codes of ethics<br><br>Continuing competence<br><br>Discipline and conduct<br><br>Governance<br><br>Reform<br><br>Regulatory models<br><br>Trade and mobility agreements<br><br>Other | Registered Nurses<br><br>Licensed Practical Nurses<br><br>Registered Psychiatric Nurses<br><br>Nurse Practitioners | National<br><br>Provincial or territorial | |
